# Correlation of mRENAL score with clinical outcomes after CT-guided renal cell carcinoma cryoablation: A retrospective observational study

**DOI:** 10.5339/qmj.2026.2

**Published:** 2026-03-03

**Authors:** Mohammad Elhissi, Eiman Musa, Yaman M. Alahmad, Aminah Alaani, Ahmed Omar, Ayman Elmajdoub, Khalid Al-Rumaihi, Husain Alturkistani, Mohamad Alhoda Mohamad Alahmad, Ali Barah

**Affiliations:** 1 Department of Clinical Imaging, Hamad Medical Corporation, Doha, Qatar; 2 Department of Urology, Hamad Medical Corporation, Doha, Qatar; 3 Department of Radiology and Medical Imaging, College of Medicine, King Saud University, Riyadh, Saudi Arabia; 4 Department of Internal Medicine, University of Kansas Medical Center, Kansas City, Kansas, United States *Email: yalahmad1@hamad.qa

**Keywords:** Renal cell carcinoma, nephrometry score, cryoablation, neoplasm recurrence, interventional radiology, CT-guided renal cell carcinoma

## Abstract

**Background and objective:**

CT-guided cryoablation (CRA) has emerged as a safe and effective minimally invasive treatment for renal cell carcinoma (RCC), though outcomes can vary. The mRENAL score may help to predict clinical outcomes. The main objective of this study was to evaluate the correlation between mRENAL score and clinical outcomes in RCC cases treated by CT-CRA.

**Methodology:**

This was an observational, retrospective, single-center study that included CT-CRA procedures performed on RCCs between 2017 and 2023. Tumors were classified into three categories based on the mRENAL score: low, intermediate, and high risk. We employed the Firth logistic regression for multivariate analysis due to the small sample size and rare outcome events.

**Results:**

Our data included 53 cases, with a mean age of 53 ± 12 years. Technical success was achieved in all cases. Four cases had disease recurrence at the site of previously treated RCC (4/42, 9.5%), as 11 cases lost follow-up imaging. The Firth logistic regression analysis revealed that increasing mRENAL score was associated with a higher risk of recurrence (adjusted odds ratio = 0.42 [95% CI, 0.19–0.95]; *P* = 0.038). This trend towards statistical significance implies that for every 1-point increase in mRENAL score, the odds of clinical success decrease by at least 5% with over 95% confidence. No procedure-related mortality was observed.

**Conclusion:**

Our analysis demonstrates that the mRENAL score may help predict clinical outcomes, with lower mRENAL scores associated with higher clinical success rates following CT-CRA of RCC.

## 1. INTRODUCTION

Renal cancer is among the most prevalent cancers in the world, accounting for 2.2% of all cancers in 2020, leading to 1.8% of all cancer-related mortality.^1^ In the Middle East and North Africa (MENA) region, renal cancer incidence increased by 98% between 1990 and 2019, reaching 15,700 new cases and causing 6000 cancer-related deaths annually. The rising renal cancer burden in the MENA region is largely attributed to increased incidental detection of renal masses through cross-sectional imaging, driven by improvements in socio-demographic indices in most MENA countries.^2^

Subsequently, multiple surgical and percutaneous treatment options have been introduced to manage renal tumors. Percutaneous ablative techniques such as microwave ablation (MWA), radiofrequency ablation (RFA), and cryoablation (CRA) have been proven to be safe and effective treatments for renal masses, with progression-free and overall survival rates comparable to partial nephrectomy.^3^ CRA has demonstrated superiority over other ablative modalities such as RFA and MWA in renal tumor treatment.^4^

The 2022 European Association of Urology (EAU) guidelines conditionally recommend percutaneous CRA for patients with T1a renal cell carcinoma (RCC) tumors (<4 cm), particularly for elderly individuals and individuals with comorbidities.^5^

Despite its advantages, CT-guided cryoablation (CT-CRA) encounters several technical challenges, some of which can be mitigated by ancillary protective techniques. The modified RENAL (mRENAL) score, which incorporates Radius, Exophytic/Endophytic, Nearness to the collecting system, Anterior/posterior location, and Location relative to renal polar lines, was originally developed to assess partial nephrectomy complexity.^6^

In the literature, limited research has evaluated the predictive value of the mRENAL score on clinical success, defined as the absence of tumor residual/recurrence, for RCC treated by CT-CRA.^7^ However, pre-procedure assessment for CT-CRA is critical to predict the clinical expectations. The main objective of this study was to evaluate the correlation between mRENAL score and clinical outcomes in RCC treated by CT-CRA.

## 2. METHODOLOGY

This is an observational, retrospective, single-center study conducted on patients who underwent CT-CRA of RCC. Approval to conduct the study was obtained from our institutional review board with waived informed consent (MRC-01-25-431 issued on May 27, 2025).

### 2.1 Patient population

**Figure 1:**
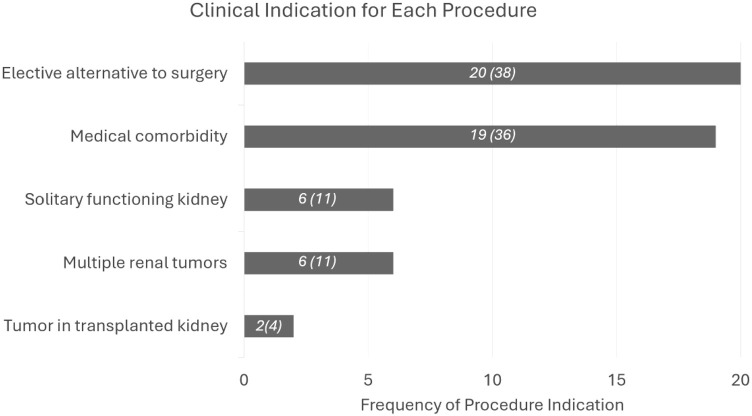
Clinical indications for CT-guided cryoablation in our sample.

A retrospective review of institutional data from 2017 to 2023 identified 53 cases (49 patients, 4 had the procedure twice) who underwent CT-CRA. Indications for CT-CRA were determined by a multidisciplinary uro-oncological board (Figure 1). All sampled patients underwent cross-sectional imaging, contrast-enhanced either CT or MRI, which demonstrated a solid renal mass.

### 2.2 Inclusion criteria

- Adult patients with stage T1a or T1b biopsy-proven or highly suspected image patterns for RCC.
- Patients suitable for CT-CRA.
- Patients with available pre- and post-procedural cross-sectional imaging and a life expectancy exceeding 3 months.

### 2.3 Exclusion criteria

- A history of ipsilateral total or partial nephrectomy.
- A history of metastatic disease.
- A history of major renal vascular invasion.
- An Eastern Cooperative Oncology Group (ECOG) score greater than three.^8^

All patients provided informed consent after a detailed explanation of the procedure, potential complications, and expected outcomes. All treatments were performed by the same interventional radiologist, who has over 10 years of experience with imaging-guided thermal ablation.

### 2.4 Tumor characteristics

Pre- and post-procedure abdomen/renal contrast-enhanced CT or MRI studies available in the Picture Archiving and Communication System (PACS) were retrospectively reviewed by a body imaging radiologist. The mRENAL score was calculated based on the parameters outlined in Table 1.^9^ The anterior or posterior tumor location does not contribute points to the mRENAL score but is documented descriptively to aid in CT-CRA planning. Tumor complexity was categorized into three risk grades based on the cumulative mRENAL score. Tumors with a score ≤6 were classified as low risk, those with scores 7 to 9 as intermediate risk, and those with scores ≥10 as high risk.

### 2.5 Procedural technique

Percutaneous CRA was performed in standard fashion under extended local sterility, using the Endocare system under CT-guidance (Endocare Inc., Irvine, CA).^10^

Following our institutional protocol, ultrasound-guided biopsy of the renal tumor was routinely performed immediately before CRA. Then, a 0.018-inch 3 × 2-mm Tornado micro-coil (Cook Medical, Bloomington, IN) was pushed and placed inside the tumor through the cannula. Patients who underwent previous biopsy were bypassed from this step, and no coil marking was placed.

**Figure 2:**
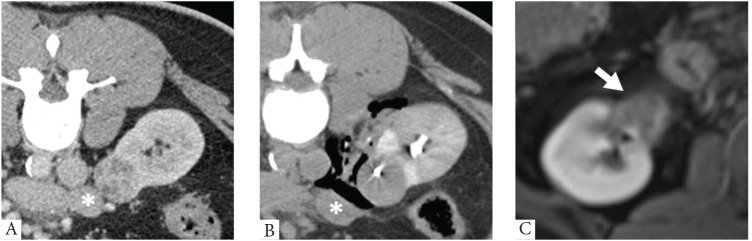
Ancillary protective maneuver using carbo-dissection in low-risk mRENAL score. (A) Intra-procedural contrast-enhanced CT showing an anterior exophytic right renal mass in contact with small bowel loops (white asterisk). (B) Post-treatment showing carbo-dissection displacing the right peri-renal structures. A radio-dense intra-tumoral coil marker inserted before treatment is also seen (C) 1-year post treatment follow-up of MRI showed no enhancement at the site of cryoablation (white arrow), suggesting the absence of disease recurrence.

Ancillary protective maneuvers, including hydro-dissection, carbo-dissection, iatrogenic pneumothorax, and ureteral stent insertion, were performed when there was a potential risk of organ injury due to proximity to the ablation zone or the presence of vulnerable structures along the path of the cryoprobes (Figure 2).

### 2.6 Operational definitions

Using the terminology suggested by Ahmed et al., in which residual unablated tumor was defined as an enhancement in the ablation cavity detected at the first follow-up after the procedure, or if residual tumor was suspected at the first follow-up scan and confirmed at the next scan or by biopsy.^11^

Local tumor recurrence was defined as nodular enhancement in the ablation cavity found in follow-up after the initial scan showed no residual tumor.

We defined clinical success as the absence of tumor residual or recurrence over the available follow-up studies.

### 2.7 Follow-up and data collection

**Figure 3:**
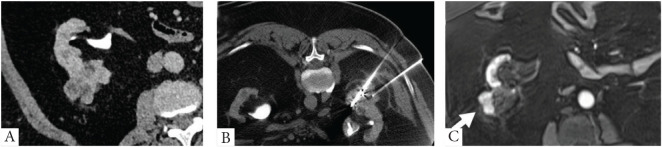
Recurrence in an Intermediate risk mRENAL score. (A) Pre-procedure contrast-enhanced CT abdomen and pelvis showing an exophytic interpolar enhanced right renal mass. (B) Intra-procedure CT showing two cryoprobes. A post-biopsy peri-renal hematoma is also noted. (C) 1.5-year post-procedural MRI follow-up shows an area of post-contrast enhancement (white arrow) concerning recurrence.

Follow-up data were collected until December 2024. Standard follow-up diagnostics included multiphase contrast-enhanced CT or MRI of the kidneys performed at 3-, 6-, and 12-month post-CT-CRA, followed by annual imaging. Patients with recurrent diseases (Figure 3) were offered further evaluation and management, including active surveillance or additional CT-CRA for confirmed incomplete response.

## 3. RESULTS

A total of 53 CT-CRA procedures were performed in 49 patients between 2017 and 2023. The mean patient age was 53 ± 12 years, with a male-to-female ratio of 2 (39) to 1 (14). The mean tumor radius was 2.9 ± 1.5 cm. Regarding tumor complexity, (30/53, 56%) tumors were classified as low risk (mRENAL score ≤ 6), (15/53, 30%) as intermediate risk (mRENAL score 7–9), and (7, 13%) as high risk (mRENAL score ≥ 10). The mean number of cryoprobes used per procedure was 2 ± 1. Ancillary protective techniques were employed in (12/53, 23%) cases: carbo-dissection in six cases, hydro-dissection in four cases, ureteral stenting in a case, and iatrogenic pneumothorax in a single case. There was a significant correlation between the need for protective maneuvers and the anatomical location of the lesions (*P* < 0.05).

**Figure 4:**
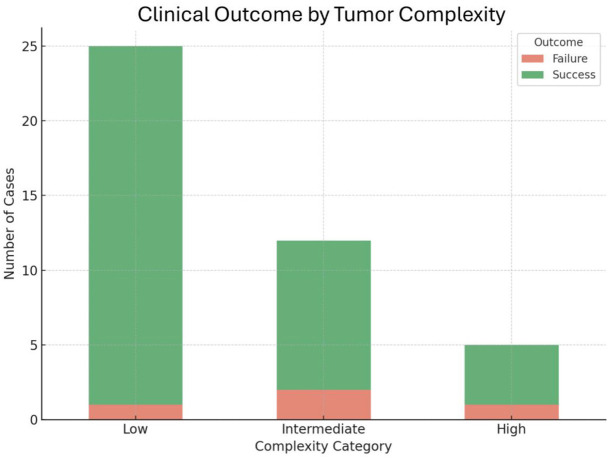
Clinical outcomes by renal cell carcinoma complexity based on mRENAL score.

Residual tumor was identified at the first imaging follow-up in none of the cases. During the follow-up period, local tumor recurrence was observed in four (4/42, 9.5%) cases, as (11/53) cases lost follow-up imaging and clinical outcomes could not be determined. The median time to recurrence was 23 months (range: 5–44 months). Management of recurrent diseases included repeat CRA in two cases, surgery in a single case, and surveillance in another case. Distribution of outcomes varied according to tumor complexity: one case of low-risk tumors, two cases of intermediate-risk tumors, and one case of high-risk tumors demonstrated recurrences. Firth logistic regression analysis revealed that increasing mRENAL score was associated with a higher risk of recurrence (adjusted odds ratio = 0.42 [95% CI, 0.19–0.95]; *P* = 0.038). This trend towards statistical significance implies that for every 1-point increase in mRENAL score, the odds of clinical success decrease by at least 5% with over 95% confidence (Figure 4). Table 2 displays the tumor characteristics of the four cases that had disease recurrence.

Complications occurred in two cases, presenting as a large post-ablation hematoma that necessitated blood transfusion in one patient and ureteral stricture in the other patient. The mean hospitalization duration was 2 ± 1 days. No procedure-related mortality was observed.

## 4. DISCUSSION

The results of percutaneous RCC ablation under CT-guidance are influenced by multiple factors, primarily determined by the safe access to the target and the accurate intra-tumoral insertion of the probes.^12^ In this context, the mRENAL score system, adapted from the surgical RENAL score system, has been specifically designed to classify the risk of renal tumor ablation performed under CT-guidance, offering a distinct classification for tumor size.^9^ However, there is a degree of controversy in the literature regarding the correlation between mRENAL score and the outcomes of CT-CRA.^7^

The findings of our study align with the reported significant correlation between the grade of mRENAL score and the clinical success outcomes of CT-CRA.^7^ In line with this, four of our patients experienced tumor recurrence, which represents a tumor progression rate of 9.5%; one was graded high risk mRENAL, two were intermediate risk, and one was graded as low risk.

Our findings support the role of the mRENAL score in predicting oncologic outcomes after percutaneous CRA. Other clinically relevant scoring systems in this context include the PADUA score, which was originally developed to classify surgical complexity and has been validated as a predictor of complications following partial nephrectomy.^13^ Similarly, the (MC)^2^ score was specifically designed to stratify the risk of major complications in percutaneous renal CRA by incorporating tumor size, location, and patient comorbidities.^14^ Recent multicenter data suggest that none of the scores were predictive of primary technique effectiveness.^14^

Tumor size has received significant attention in the literature due to its prognostic implications for survival and disease progression. Current guidelines suggest the application of thermal ablation techniques in renal tumors with a size measuring ≤3 cm, while remaining skeptical concerning larger tumors.^15^ Meanwhile, numerous studies have demonstrated the efficacy and safety of CRA in the treatment of renal tumors up to 4 cm.^16^ It is expected that the larger the tumor, the higher the number of cryoprobes needed to achieve complete treatment, which in turn increases the risk of cryoinjury to adjacent organs. This makes the precise intra-tumoral placement of these needles more challenging and critical for treatment success. To address this, we routinely placed intra-tumoral coil markers as landmarks to aid in the ballistic insertion of the cryoprobes within the tumor, as presented previously in Figure 2. This practice has proven effective in improving needle insertion accuracy as demonstrated previously in CT-guided MWA and RFA.^17^ However, despite using coil markers in such tumors, we observed tumor recurrence in four cases. Notably, the average largest tumor radius was slightly greater in these cases compared to the rest of the sample (3.4 cm vs. 2.7 cm), which may have contributed to tumor recurrence.

In addition to tumor size, the endophytic growth pattern of tumors posed challenges during CT-CRA. It is well-established that RCC is completely endophytic, with an intact overlying renal cortex, which presents technical challenges for surgical eradication due to difficulty with tumor margin visualization.^17^ While studies have demonstrated that CT-CRA is a viable alternative to surgery for such tumors, achieving a complete response while minimizing the risk of collecting system injury remains challenging.^18^^,^^19^ To address this, some authors suggested ancillary protective techniques such as a trans-arterial lipiodol injection to enhance tumor delineation, antegrade or retrograde pyeloperfusion to protect the collecting system and renal artery balloon insertion to mitigate the heat-sink effect in case of thermal ablation.^20^^–^^22^ In our study, 19% of tumors were completely endophytic, while 42% had at least 50% of their volume located between the polar lines or crossing the renal midline. Despite that, we had a high clinical success of 90%. On the other hand, two of our endophytic cases, located less than 4 mm from the collecting system, showed recurrence due to the challenges in achieving complete ablation.

### 4.1 Clinical implications

The main target of CT-CRA is to achieve destruction of the RCC while ensuring a safe margin of 5 to 10 mm to minimize the risk of adjacent organs. Given this, the ancillary protective maneuvers are crucial in overcoming most of the challenges during CT-CRA of RCC.^23^ In our series, protective techniques were employed in 12 cases, where they contributed significantly to obtaining successful outcomes. We observed a significant correlation between the need for protective maneuvers and the anatomical location of the tumors, particularly in those located anteriorly and above or below the polar lines. This finding highlights the unique challenges posed by these locations, likely due to their proximity to bowels, which necessitate additional protective strategies. Interestingly, the lower mRENAL category was significantly associated with the use of protective techniques, such as pneumo- or hydro-dissection. None of the cases graded as high mRENAL score required these techniques. This finding limits the utility of the mRENAL scoring system in predicting the need for additional protective techniques during CT-CRA.

In practice, we faced difficulties in accessing the RCC located anteriorly. Although the anterior tumors are included in the mRENAL score as an independent parameter, it does not contribute additional points to the final score and are only categorized as distinct from the posterior tumor.^9^ Anterior tumors present several challenges for CT-CRA, primarily due to limited safe access to tumors by cryoprobes in patients, mostly in the prone position. This limitation complicates optimal intra-tumoral cryoprobes placement and may compromise clinical outcomes. Moreover, anterior tumors are frequently located in proximity to organs and bowels, increasing the risk of cryoinjury and reducing clinical success. To mitigate these risks, pneumo- and hydro-dissection are commonly employed in such cases.^23^ In our series, ancillary protective techniques were employed in 12 cases, where they contributed significantly to obtaining successful outcomes. We observed a significant correlation between the need for protective maneuvers and the anatomical location of the tumors, particularly in those located anteriorly and above or below the polar lines. This finding highlights the unique challenges posed by these locations, likely due to their proximity to bowels, which necessitate additional protective strategies.

### 4.2 Limitations

Our study has several limitations. First, it is a retrospective review of a single institution’s experience with CT-CRA of renal tumors. Second, there is a potential selection bias in the referral process by the urology-oncology multidisciplinary team for consideration of CT-CRA. Third, the study includes a relatively small cohort of patients, with a mean follow-up period of over 2 years, and missing data for some variables. Lastly, the data extends over 6 years period, during which there were inevitable changes in operator experience and advancements in ablation technology.

## 5. CONCLUSION

The mRENAL score offers a potential predictive role for CT-CRA clinical success on RCC cases. It is a valid tool to be utilized in assessing outcomes before CT-CRA.

## CONFLICT OF INTEREST

The authors declare that there is no conflict of interest.

## Figures and Tables

**Table 1.** mRENAL score.

Characteristic	1-point	2-points	3-points
mR (maximum diameter)	≤ 3 cm	>3 cm and ≤ 4 cm	>4 cm
E (% exophytic)	≥ 50%	<50%	Entirely endophytic
N (nearness of tumor to collecting system)	≥ 7 mm	>4 mm and <7 mm	≤ 4 mm
L (location relative to polar lines)	Entirely above the upper pole or below the lower pole)	The lesion crosses the polar line	Fifty percent of mass is across the polar line, or mass is entirely between polar lines, or mass crosses the axial renal midline.
A (Anterior/posterior)	No points		

**Table 2.** Tumor characteristics based on mRENAL scores of the four cases that had renal tumor recurrence after cryoablation.

Tumor #	mR	E	N	L	mRENAL score	Ancillary maneuver
1	2	2	3	2	9	Yes
2	1	3	3	3	10	No
3	1	2	1	1	5	No
4	2	3	1	3	9	No
